# Clinical and epidemiological aspects of Delta and Gamma SARS-CoV-2 variant of concern from the western Brazilian Amazon

**DOI:** 10.1590/0074-02760220155

**Published:** 2023-01-20

**Authors:** Gabriella Sgorlon, Jackson Alves da Silva Queiroz, Tárcio Peixoto Roca, Ana Maisa Passos da Silva, Nadson Willian Felipe Gasparelo, Karolaine Santos Teixeira, Andreia Souza da Nóbrega Oliveira, Aline Linhares Ferreira de Melo Mendonça, Adriana Cristina Salvador Maia, Soraya dos Santos Pereira, Flávia Serrano Batista, Juan Miguel Villalobos Salcedo, Rita de Cassia Pontello Rampazzo, Paola Cristina Resende, Marilda Mendonça Siqueira, Felipe Gomes Naveca, Deusilene Vieira

**Affiliations:** 1 Fundação Oswaldo Cruz, Laboratório de Virologia Molecular, Porto Velho, RO, Brasil; 2 Universidade Federal de Rondônia, Programa de Pós-Graduação em Biologia Experimental, Porto Velho, RO, Brasil; 3 Fundação Oswaldo Cruz, Instituto Oswaldo Cruz, Laboratório de Hepatites Virais, Rio de Janeiro, RJ, Brasil; 4 Laboratório Central de Saúde Pública do Estado de Rondônia, Porto Velho, RO, Brasil; 5 Agência Estadual de Vigilância em Saúde de Rondônia, Coordenação Estadual da Covid-19, Porto Velho, RO, Brasil; 6 Instituto de Biologia Molecular do Paraná, Curitiba, PR, Brasil; 7 Fundação Oswaldo Cruz, Instituto Oswaldo Cruz, Laboratório de Vírus Respiratórios e do Sarampo, Rio de Janeiro, RJ, Brasil; 8 Fundação Oswaldo Cruz, Instituto Leônidas e Maria Deane, Laboratório de Virologia, Manaus, AM, Brasil

**Keywords:** SARS CoV-2, variant of concern, Gamma, Delta, genomic surveillance

## Abstract

**BACKGROUND:**

The emergence of severe acute respiratory syndrome coronavirus 2 (SARS-CoV-2) variants has become a major concern contributing to increased morbidity and mortality worldwide.

**OBJECTIVES:**

Here we describe the replacement of the Gamma variant of concern (VOC) with Delta in the western Brazilian Amazon.

**METHODS:**

In this study, we analysed 540 SARS-CoV-2 positive samples determined by qualitative real-time RT-PCR selected in the state of Rondônia between June and December 2021. The positive cohort was sequenced through next-generation sequencing (NGS) and each sample was quantified using real-time RT-qPCR, the whole genome sequence was obtained, SARS-CoV-2 lineages were classified using the system Pango and the maximum likelihood (ML) method was used to conduct phylogenetic analyses.

**FINDINGS:**

A total of 540 high-quality genomes were obtained, where the Delta VOC showed the highest prevalence making up 72%, with strain AY.43 being the most abundant, while the Gamma VOC was present in 28%, where the P.1 strain was the most frequent. In this study population, only 32.96% (178/540) had completed the vaccination schedule.

**MAIN CONCLUSIONS:**

This study highlighted the presence of Gamma and Delta variants of SARS-CoV-2 in RO. Furthermore, we observed the replacement of the Gamma VOC with the Delta VOC and its lineages.

Coronavirus disease (COVID-19) is caused by severe acute respiratory syndrome coronavirus 2 (SARS-CoV-2) and remains a worldwide concern nearly 2 years after a pandemic was declared.^1^ This virus presents a high rate of transmissibility, thus acquiring many mutations that favour the emergence of variants of concern (VOC), which in turn may be characterised by increased infectivity and/or the potential for immune evasion, especially that of neutralising anti-SARS-CoV-2 antibodies. The Gamma (P.1), Delta (B.1.617.2 and AY.*), Alpha (B.1.1.7 and Q.*), Beta (B.1.351) and, recently, Omicron (B.1.1.529 and BA.*) VOCs have shown the highest impact on public health to date.^2^

The Gamma VOC was detected in December 2020 in northern Brazil, more precisely in Manaus, which has been identified as the beginning of the second wave of COVID-19 in the country.^3^ The Delta VOC was first described in India, in October 2020, and was identified in April 2021 when the first case occurred in southern Brazil, linked to a person who had travelled to Asia.^4^ ^,^ ^5^ ^)^ Since then, the high rate of transmissibility of these variants has permitted the appearance of new sublineages, which is a phenomenon that is an integral part of viral evolution.^6^ ^-^ ^8^

As has happened in other countries, the North Region of Brazil has shown a high incidence and cumulative mortality rate since these VOCs were originally detected. The state of Rondônia (RO) presented the highest mortality rate in the region, according to the Ministry of Health, which highlights the importance of implementing genomic surveillance.^9^ The objective of this study was to describe the profile of SARS-CoV-2 variants in the western Brazilian Amazon region between June and December 2021.

## MATERIALS AND METHODS

*Ethical aspects and study site -* This study was conducted at Fiocruz/RO, under the authorisation of the FIOCRUZ COVID-19 Genomics Surveillance Network of the Brazilian Ministry of Health and was approved by the Research Ethics Committee of the Centro de Pesquisa em Medicina Tropical de Rondônia (protocol 4.000.086). All experiments were performed in accordance with relevant guidelines and regulations and was exempted from informed consent requirements owing to its retrospective design.

*Biological samples and epidemiological data -* SARS-CoV-2 positive individuals were conveniently sampled in primary health units and reference centres in different municipalities of RO. Laboratory diagnosis was performed by RT-qPCR at Laboratório Central de Saúde Pública de Rondônia (LACEN/RO) using the One Step/COVID-19 Kit [Instituto de Biologia Molecular do Paraná (IBMP), Curitiba, Brazil], and a total of 540 samples with cycle threshold (Ct) values < 25 for the viral target were selected for the study. Epidemiological data and vaccination status were collected from medical records in Gerenciador de Ambiente Laboratorial (GAL/RO), Sistema de Informação da Vigilância Epidemiológica da Gripe (SIVEP-Gripe) and E-SUS databases. 

*Complete genome sequencing of SARS-CoV-2 -* Complete genome sequencing of SARS-CoV-2 samples with Ct values < 25, based on qualitative assays, were selected to allow for high genomic coverage. Nucleotide sequencing was performed using Illumina MiSeq or NextSeq platforms and the COVIDSEQ Kit (Illumina, San Diego, USA).^10^

*Data acquisition and maximum likelihood (ML) phylogeny -* High-quality (< 1% of N) complete genomes (> 29 kb) of SARS-CoV-2 (n = 544, corresponding to two representatives for each states of Brazil and lineage found) were retrieved from the GISAID EpiCoV database^11^ on December 22, 2021 and the sequences were aligned using MAFFT v.7.487.^12^ The ML method was adopted using IQ-TREE v.2.1.3^13^ and the best-fitting nucleotide substitution model was GTR+G+I using the ModelFinder tool.^14^ Ultrafast bootstrap with 1,000 replicates was used to obtain branch support values. The tree was visualised and edited with FigTree v.1.4.4.^15^ SARS-CoV-2 genomes were classified into lineages using the available software Pangolin^7^ and mutations were analysed with Nextclade Beta.^16^

*Nucleic acid isolation and RT-qPCR -* Sample quantification was performed in the Laboratório de Virologia Molecular (Fiocruz/RO) where viral RNA was extracted from 140 µL of pooled swab samples using a QIAamp^®^ Viral RNA Mini Kit (QIAGEN, Hilden, Germany), according to the manufacturer’s instructions. Viral load was determined using 5 μL of this extracted viral RNA using the Multiplex One-Step RT-qPCR assay for detection of SARS-CoV-2, as developed by Queiroz et al.^17^

*Statistical analysis -* Descriptive analyses were represented through central tendency and dispersion measurements. The chi-square test was used for statistical inference with a significance level of 5% (p < 0.05). Statistical analysis was performed and graphics were generated using the software R v4.0.3.

## RESULTS

**Fig. 1:**
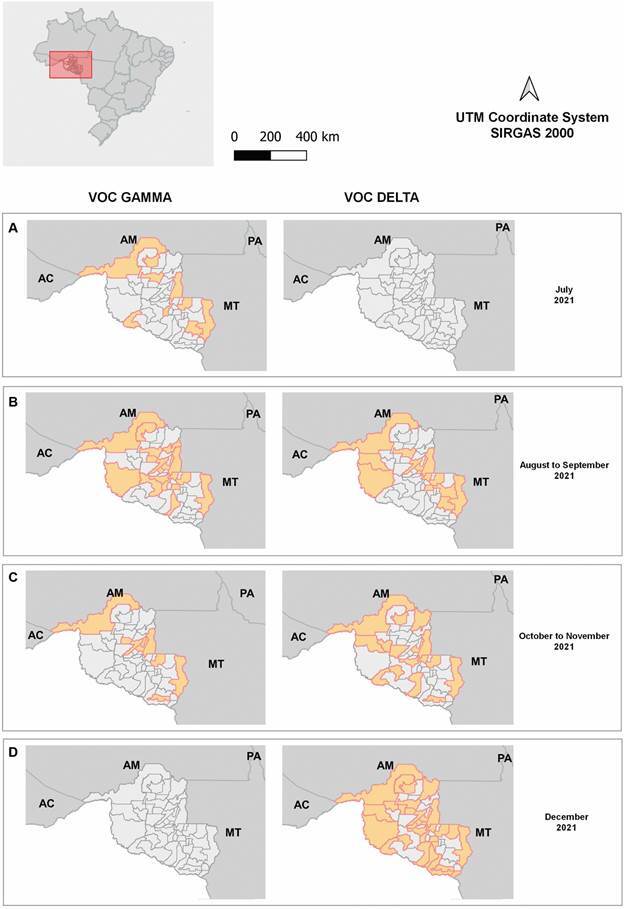
temporal substitution of the Gamma variant of concern (VOC) for the Delta VOC from July to December 2021 in municipalities of the state of Rondônia. A) July: absence of Delta VOC detection; B) August-September: insertion of the Delta VOC followed by reduction of the Gamma VOC; C) October-November: increase in the Delta VOC, indicating the overlap profile in relation to the Gamma VOC; D) December: only Delta VOC was detected. Brazilian states: Acre (AC), Amazonas (AM), Pará (PA), Mato Grosso (MT).

A total of 540 samples from 36 municipalities in RO were selected and sequenced (< 1% N, or nucleotides not identified). The Delta variant was prevalent, accounting for 72% (390/540) of the characterised sequences, while the Gamma variant was found in 28% (150/540) of cases (Fig. 1).

**Fig. 2:**
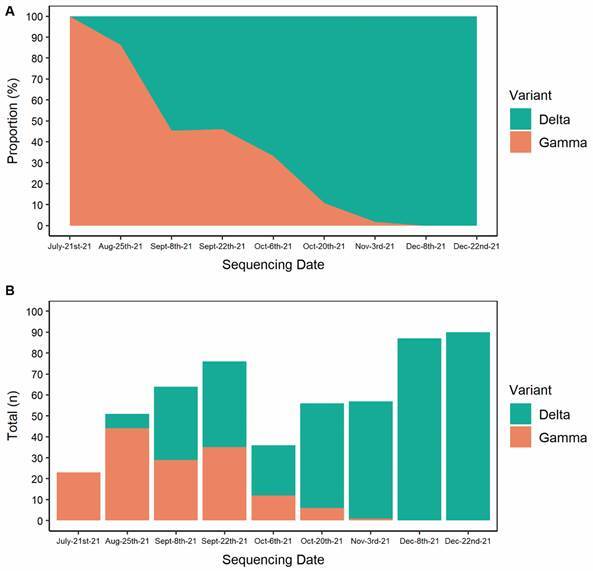
area plot (A) and bar plot (B) representing the relative and absolute proportion of the Gamma and Delta variants of concern (VOCs) over time, respectively.

Fig. 2 represents the temporal dynamics of infections for the identified variants, where it is possible to observe the increase in the proportion of the Delta variant in relation to Gamma.

**Fig. 3:**
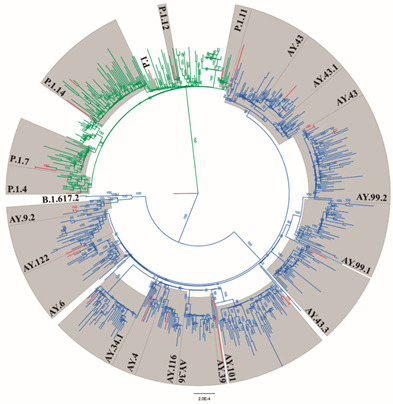
maximum likelihood phylogenetic tree including representatives isolated from the severe acute respiratory syndrome coronavirus 2 (SARS-CoV-2) Delta and Gamma variant study and 544 full genome sequences retrieved from GISAID. Samples are in the red branches. Delta and Gamma variants are in blue and green clades, respectively. Bootstrap values are in the branches.

The ML phylogeny regarding the classification and distribution of the major clades of Delta and Gamma variants is shown in Fig. 3. In addition to the parent variant B.1.617.2 (45/390), other Delta lineages were found, the most prevalent being AY.43 (179/390), followed by AY.99.2 (139/390), AY.9.2 (10/390), AY.101 (4/390), AY.4 (3/390), AY.122 (2/390), AY.6 (1/390), AY.34.1.1 (1/390), AY.36 (1/390), AY.39 (1/390), AY.43.1 (1/390), AY.46.3 (1/390), AY.99.1 (1/390) and AY.116 (1/390). Among the Gamma variants, the P.1 line of origin was most frequently encountered (78/150), followed by the subvariants P.1.4 (41/150), P.1.7 (21/150), P.1.14 (8/150), P.1.11 (1/150) and P.1.12 (1/150). 

Individuals infected with the Delta VOC showed a median age of 37 years old [standard deviation (SD) 16.96], with ages ranging from 6 to 86 years old and 51% (200/390) were female while 49% (190/390) were male. The Gamma VOC population showed a median age of 34 years old (SD 18.6), ranging from 7 to 90 years old, and 56% (84/150) were female while 44% (66/150) were male. 

In this cohort, 32.96% (178/540) were considered to be fully vaccinated (at least 15 days after the second or third dose of the vaccine at the time of sample collection), 41.66% (225/540) were partially immunised (at least 15 days after the first dose of the vaccine at the time of sample collection), and 25.37% (137/540) were unvaccinated (Table). The complete immunisation rate among individuals over 50 years of age was 46.62% (83/178), 39.88% (71/178) among individuals aged 30-50 years and 13.48% (24/178) in individuals under 30 years of age.

**TABLE:** Distribution of identified lineages in relation to the immunisation profile of the study population

Variant	Lineage	Complete immunisation (n = 178)	%	Partial immunisation (n = 225)	%	No immunisation (n = 137)	%	Total (n = 540)
Delta (n = 390)	B.1.617.2	9	5.1	24	10.7	12	8.8	45
	AY.4	1	0.6	2	0.9	0	0.0	3
	AY.6	0	0.0	0	0.0	1	0.7	1
	AY.9.2	6	3.4	2	0.9	2	1.5	10
	AY.34.1.1	1	0.6	0	0.0	0	0.0	1
	AY.36	0	0.0	0	0.0	1	0.7	1
	AY.39	0	0.0	0	0.0	1	0.7	1
	AY.43	59	33.1	75	33.3	45	32.8	179
	AY.43.1	0	0.0	1	0.4	0	0.0	1
	AY.46.3	1	0.6	0	0.0	0	0.0	1
	AY.99.1	1	0.6	0	0.0	0	0.0	1
	AY.99.2	74	41.6	36	16.0	29	21.2	139
	AY.101	0	0.0	1	0.4	3	2.2	4
	AY.116	0	0.0	1	0.4	0	0.0	1
	AY.122	0	0.0	0	0.0	2	1.5	2
Gamma (n = 150)	P.1	11	6.2	42	18.7	25	18.2	78
	P.1.4	6	3.4	25	11.1	10	7.3	41
	P.1.7	5	2.8	12	5.3	4	2.9	21
	P.1.11	0	0.0	1	0.4	0	0.0	1
	P.1.12	0	0.0	0	0.0	1	0.7	1
	P.1.14	4	2.2	3	1.3	1	0.7	8
Hospitalisation (n = 5)	B.1.617.2	0	0.0	1	0.5	0	0.0	1
	P.1	0	0.0	1	0.5	0	0.0	1
	P.1.4	1	1.0	0	0.0	2	1.7	3
Death (n = 4)	AY.43	0	0.6	1	0.0	0	0.0	1
	P.1	0	0.0	2	1.0	1	0.8	3

Percentage of Delta (AY.*+B.1.617.2) and Gamma (P.1.*+P.1) strains identified in relation to the immunisation profile of the study population. Individuals were classified into three distinct groups: complete immunisation (after receiving the second or third dose of the vaccine), partial immunisation (after receiving the first dose of the vaccine) or no immunisation.

Among the nine hospitalised individuals, two children under 10 years of age (7 and 8 years old) were not vaccinated and did not present comorbidities, one of whom evolved to death. 

The other three individuals that died were elderly, partially immunised and presented comorbidities. There were no recorded deaths among fully immunised individuals.

The symptoms were similar in the two groups, with 61% (329/540) of individuals presenting cough, 54% (297/540) fever, 54% (299/540) headache, 36% (196/540) sore throat and 35% (190/540) runny nose. The least frequently reported symptoms among the individuals were 18% (98/540) presenting olfactory disorders, 16% (86/540) reporting taste disorders and 12% exhibiting (63/540) dyspnoea; 2% (10/540) of patients were asymptomatic. 

**Fig. 4:**
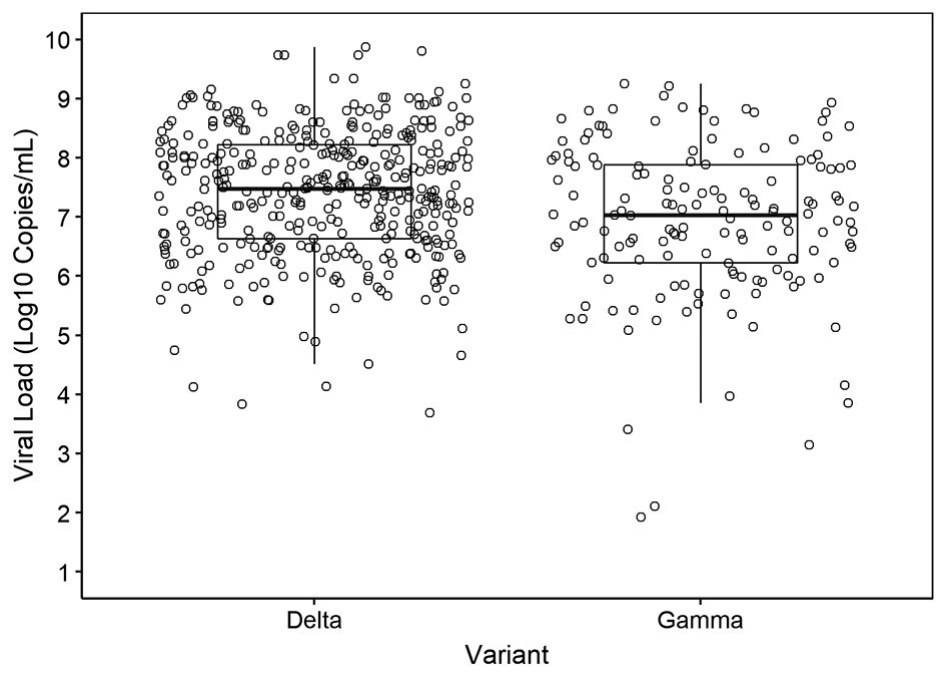
viral load profile of samples characterised as positive for severe acute respiratory syndrome coronavirus 2 (SARS-CoV-2) Delta or Gamma variants based on RT-qPCR.

We observed no difference regarding the viral load when individuals with Gamma or Delta infections were compared; an interquartile median of 7.47 Log10 copies/mL (min:3.68 max:9.87) for the Delta VOC and 7.03 Log10 copies/mL (min:1.92 max:9.24) for the Gamma VOC (Fig. 4).

## DISCUSSION

In this study, we analysed socio-demographic data, viral load and SARS-CoV-2 circulating lineages in the second half of 2021 in RO, located in the northern region of Brazil. Our analyses revealed that the Gamma VOC and its subvariants were predominant between July and August 2021, the period preceding the entry of the Delta VOC and its subvariants. 

The Gamma VOC was responsible for rapidly spreading waves of infections in Brazil, making it the most prevalent variant from January through August 2021,^18^ ^,^ ^19^ possibly justified by its high transmissibility and partial immune evasion, as shown by reinfection cases.^20^ However, this scenario changed after the introduction of the Delta VOC with the first confirmed case in RO on August 3, 2021, leading to a gradual increase in cases as described in this study. The incidence of SARS-CoV-2 in the state decreased between June and October, from 237.8 to 22.8 cases per 100,000 inhabitants; however, in November and December, there was a significant increase where the incidence rates recorded were 80.7 and 126.7, respectively, with the highest numbers in relation to the other states in the northern region of the country.^9^ ^,^ ^21^ ^-^ ^27^

Published data demonstrate rapid growth and dissemination of this variant on a global scale,^28^ ^-^ ^31^ being about two times more transmissible than the original Wuhan SARS-CoV-2 strain.^32^ ^-^ ^34^ Furthermore, a comparative study suggests a clear competitive advantage of the Delta variant over the Alpha, Beta and Gamma variants, showing an estimated increase in the number of effective replications by about 55, 60 and 34%, respectively.^33^

Mutations in the viral genome allowed for the emergence of sublineages relative to the original strain. The Delta subvariant AY.99.2 was predominant in Brazil between August and December 2021^35^; however, in this study a higher prevalence of AY.43 was observed during the same period. Studies have shown that the posterior division of this lineage into AY.43.1 and AY.43.2 originated in Brazil.^36^ In addition, the subvariants AY.4.2 and AY.1, also described as “Delta plus”, both associated with an increased number of Delta cases in several countries, were not detected.^37^ Regarding the Gamma lineage, we observed a higher proportion of P.1.4 than P.1.7, different from previously published data in the country, which show a higher prevalence of P.1.7 compared to P.1.4.^10^ The subvariant profile was different than that of the state of Amazonas, where besides P.1.4, there was also a predominance in the P.1.3 and P.1.6 lineages during this same time period; both states are located in the northern region of the country.^10^

On the other hand, vaccination in children between 5 and 11 years of age has been widely recommended by health agencies^38^ ^,^ ^39^ and has been demonstrated to be safe, immunogenic and effective against COVID-19.^40^ We report two cases of children in this age group, who were not vaccinated, required hospitalisation and one of whom died.

Infection of fully vaccinated individuals is a reality in COVID-19, mainly due to the emergence of new variants and mutations that may favour immune escape.^41^ ^-^ ^44^ Even with the possibility of infection after vaccination, vaccinated individuals are less likely to develop the severe form of the disease or die.^43^ Furthermore, due to the emergence of new variants, booster doses of immunisations are essential as a measure to protect against and combat the disease.^45^ ^-^ ^47^ In our study, the three adults who died were not fully vaccinated.

During June and December 2021, RO went from having 7.2 to 55.2% of the population fully immunised, and 12.6 to 67.7% of the population being partially immunised.^48^ ^)^ However, the profile of the study population demonstrated a low immunisation rate of individuals aged around 30 years old, which may reflect hesitancy towards vaccination, previously observed in the working-age population in other countries,^49^ ^,^ ^50^ as well as in Brazil.^41^

Infection of fully vaccinated individuals is a reality in COVID-19, mainly due to the emergence of new variants and mutations that may favour immune escape.^43^ ^,^ ^44^ ^,^ ^51^ ^,^ ^52^ Even with the possibility of infection after vaccination, vaccinated individuals are less likely to develop severe disease or die.^53^ In this population, only one death was observed, whose individual had only one dose of the vaccine, besides advanced age and comorbidities, not being reported cases of death in fully vaccinated individuals.

Most patients had the same triad of symptoms, including cough, fever, headache, supporting studies that report no divergent aspects of symptoms with the progressive introduction of new strains and VOCs in other countries.^54^ ^,^ ^55^ Olfactory and gustatory disturbances were observed at the start of the pandemic as the most frequent symptoms; however, in our study, these symptoms were not frequent.^56^

The constant emergence of new variants suggests the need to maintain preventive measures, such as the use of adequate masks, prioritisation of professional and educational activities, as well as avoiding unnecessary social agglomerations, in order to reduce the probability of SARS-CoV-2 evolution; we encourage vaccination, since severe cases were not seen in fully vaccinated patients within the two VOCs analysed.^48^ ^,^ ^49^

In conclusion, this study showed the replacement of the Gamma VOC with Delta and its sublineages in RO, western Brazilian Amazon, and analysed both socio-demographic and laboratorial data that may have contributed to this phenomenon. Our study further emphasises the importance of local genomic surveillance in large countries like Brazil.

*Data availability -* All the SARS-CoV-2 genomes generated and analysed in this study are available in the EpiCov database in GISAID under the following ID numbers: EPI_ISL_11112665-11112674, EPI_ISL_5030021, EPI_ISL_6575689-6575706, EPI_ISL_6575708, EPI_ISL_6575710-6575739, EPI_ISL_8623163-8623164, EPI_ISL_8623166-8623256, EPI_ISL_8623258-8623269, EPI_ISL_9414682-9414748, EPI_ISL_9414750-9414760, EPI_ISL_9414773-9414774, EPI_ISL_9636798-9636802 and EPI_ISL_9636805-9636877.

The list of accession IDs may be found in the attached file in the Supplementary data.
