# Supplementary material for: Clinical and epidemiological aspects of Delta and Gamma SARS-CoV-2 variant of concern from the western Brazilian Amazon
Source: Mem Inst Oswaldo Cruz. 2023 Jan 20;117:e220155. doi: 10.1590/0074-02760220155 (PMC9870256; doi:10.1590/0074-02760220155)
Supplement: Supplementary file 1 [file 1678-8060-mioc-117-e220155-s.pdf]

We gratefully acknowledge the following authors from the originating laboratories responsible for obtaining the specimens and the submitting laboratories where genetic sequence data were generated and shared via the GISAID Initiative.

TABLE

Laboratories and institutions that generated and shared gene sequences in the GISAID Initiative used in this study

Virus name	Accession number	Collected	Originating lab	Submitting lab	Author
hCoV-19/Wuhan/IPBC AMS-WH-01/2019	EPI_ISL_ 402123	2019-12-24	Institute of Pathogen Biology, Chinese Academy of Medical Sciences & Peking Union Medical College	Institute of Pathogen Biology, Chinese Academy of Medical Sciences & Peking Union Medical College	Ren et al.
hCoV-19/Brazil/RJ-FIOCRUZ-22118/2021	EPI_ISL_ 2443610	2021-05-05	Laboratório de Vírus Respiratórios e Sarampo, Instituto Oswaldo Cruz, Fundação Oswaldo Cruz	Laboratório de Vírus Respiratórios e Sarampo, Instituto Oswaldo Cruz, Fundação Oswaldo Cruz	Resende et al.
hCoV-19/Brazil/BA-FIOCRUZ-31255/2021	EPI_ISL_ 2863714	2021-06-18	Laboratório Central de Saúde Pública do Estado da Bahia	Laboratório de Vírus Respiratórios e Sarampo, Instituto Oswaldo Cruz, Fundação Oswaldo Cruz	Resende et al.
hCoV-19/Brazil/AM-987/2020	EPI_ISL_ 833167	2020-12-16	DB Diagnósticos do Brasil	Instituto Adolfo Lutz, Centro Interdisciplinar de Procedimentos, Laboratório Estratégico	Sacchi et al.
hCoV-19/Brazil/AM-FIOCRUZ-21841237MIA/2021	EPI_ISL_ 2777487	2021-03-04	Laboratório de Ecologia de Doenças Transmissíveis na Amazônia, Instituto Leônidas e Maria Deane - Fiocruz Amazônia	Laboratório de Ecologia de Doenças Transmissíveis na Amazônia, Instituto Leônidas e Maria Deane - Fiocruz Amazônia	Nascimento et al.
hCoV-19/Brazil/AM-FIOCRUZ-21140024DEB/2021	EPI_ISL_ 2777396	2021-01-05	Laboratório de Ecologia de Doenças Transmissíveis na Amazônia, Instituto Leônidas e Maria Deane - Fiocruz Amazônia	Laboratório de Ecologia de Doenças Transmissíveis na Amazônia, Instituto Leônidas e Maria Deane - Fiocruz Amazônia	Nascimento et al.
hCoV-19/Brazil/SP-HIAE-ID595/2021	EPI_ISL_ 3505194	2021-08-07	Laboratório de Técnicas Especiais - Hospital Israelita Albert Einstein	Laboratório de Técnicas Especiais - Hospital Israelita Albert Einstein	Amgarten et al.
hCoV-19/Brazil/ES-FIOCRUZ-40147/2021	EPI_ISL_ 3435042	2021-07-16	Laboratório Central de Saúde Pública do Estado do Espírito Santo	Laboratório de Vírus Respiratórios e Sarampo, Instituto Oswaldo Cruz, Fundação Oswaldo Cruz	Resende et al.
hCoV-19/Brazil/BA-LACEN-BA252/2021	EPI_ISL_ 3255081	2021-05-07	Laboratório Central de Saúde Pública do Estado da Bahia	Laboratório Central de Saúde Pública do Estado da Bahia	Tosta et al.
hCoV-19/Brazil/AP-FIOCRUZ-40075/2021	EPI_ISL_ 3434983	2021-06-19	Laboratório Central de Saúde Pública do Estado do Amapá	Laboratório de Vírus Respiratórios e Sarampo, Instituto Oswaldo Cruz, Fundação Oswaldo Cruz	Resende et al.
hCoV-19/Brazil/SP-HIAE-ID372/2021	EPI_ISL_ 3246184	2021-07-23	Laboratório de Técnicas Especiais - Hospital Israelita Albert Einstein	Laboratório de Técnicas Especiais - Hospital Israelita Albert Einstein	Amgarten et al.
hCoV-19/Brazil/RJ-LNN03551/2021	EPI_ISL_ 3245250	2021-07-14	Laboratório Central de Saúde Pública Noel Nutels	Laboratório de Bioinformática	Almeida et al.
hCoV-19/Brazil/PE-FIOCRUZ-IAM2275/2021	EPI_ISL_ 3046274	2021-07-01	Laboratório Central de Saúde Pública do Estado de Pernambuco	Wallau Lab em nome da Fiocruz COVID-19 Rede de Vigilância Genômica	Paiva et al.
hCoV-19/Brazil/AM-FIOCRUZ-21142276EAX/2021	EPI_ISL_ 3050641	2021-07-05	Laboratório de Ecologia de Doenças Transmissíveis na Amazônia, Instituto Leônidas e Maria Deane - Fiocruz Amazônia	Laboratório de Ecologia de Doenças Transmissíveis na Amazônia, Instituto Leônidas e Maria Deane - Fiocruz Amazônia	Nascimento et al.
hCoV-19/Brazil/SP-IAL-5338/2021	EPI_ISL_ 5863702	2021-09-20	Instituto Adolfo Lutz - Regional de Santo André	Instituto Adolfo Lutz, Centro Interdisciplinar de Procedimentos, Laboratório Estratégico	Sacchi et al.
hCoV-19/Brazil/SP-IB_121956/2021	EPI_ISL_ 5651336	2021-07-17	Unidade de Pronto Atendimento Central de Caraguatatuba	Instituto Butantan	Covas et al.
hCoV-19/Brazil/SP-NVBS2934GEN OV827790202793/2021	EPI_ISL_ 4424434	2021-07-01	DASA	DASA	Guarischi et al.
hCoV-19/Brazil/PA-FIOCRUZ-51759/2021	EPI_ISL_ 4632928	2021-08-19	Laboratório Central de Saúde Pública do Estado do Pará	Laboratório de Vírus Respiratórios e Sarampo, Instituto Oswaldo Cruz, Fundação Oswaldo Cruz	Resende et al.
hCoV-19/Brazil/SP-IB_116441/2021	EPI_ISL_ 5649102	2021-06-21	UBS Dr. Waldomiro Ferreira Neves Scrpardo	Instituto Butantan	Covas et al.
hCoV-19/Brazil/PE-FIOCRUZ-IAM3553/2021	EPI_ISL_ 3835266	2021-08-02	Laboratório Central de Saúde Pública do Estado de Pernambuco	Wallau Lab em nome da Fiocruz COVID-19 Rede de Vigilância Genômica	Paiva et al.
hCoV-19/Brazil/AM-FIOCRUZ-ILMD2100596/2 021	EPI_ISL_ 5490090	2021-07-21	Laboratório Central de Saúde Pública do Amazonas	Laboratório de Ecologia de Doenças Transmissíveis na Amazônia, Instituto Leônidas e Maria Deane - Fiocruz Amazônia	Silva et al.
hCoV-19/Brazil/AM-FIOCRUZ-21891207MSD/2021	EPI_ISL_ 4516072	2021-08-03	Laboratório Central de Saúde Pública do Amazonas	Laboratório de Ecologia de Doenças Transmissíveis na Amazônia, Instituto Leônidas e Maria Deane - Fiocruz Amazônia	Nascimento et al.
hCoV-19/Brazil/SP-NVBS8005GEN OV828204277055/2021	EPI_ISL_ 7366494	2021-10-01	DASA	DASA	Guarischi et al.
hCoV-19/Brazil/SP-IAL-5363/2021	EPI_ISL_ 5863705	2021-09-04	Instituto Adolfo Lutz - Regional de Campinas	Instituto Adolfo Lutz, Centro Interdisciplinar de Procedimentos, Laboratório Estratégico	Sacchi et al.
hCoV-19/Brazil/SP-HIAE-ID361/2021	EPI_ISL_ 3246173	2021-07-23	Laboratório de Técnicas Especiais - Hospital Israelita Albert Einstein	Laboratório de Técnicas Especiais - Hospital Israelita Albert Einstein	Amgarten et al.
hCoV-19/Brazil/SP-IB_103317/2021	EPI_ISL_ 2445049	2021-05-10	Inside diagnósticos sul parelheiros	Instituto Butantan	Covas et al.
hCoV-19/Paraguay/23 1882/2021	EPI_ISL_ 4259569	2021-08-18	Laboratório Central de Salud Pública	Laboratório Central de Salud Pública	Gonzalez et al.
hCoV-19/India/CT-ILSGS01056/20 21	EPI_ISL_ 2341881	2021-03-01	Pt. Jawahar Lal Nehru Memorial Medical College, Raipur	Institute of Life Sciences	Raghav et al.
hCoV-19/Ecuador/NIC-INSPI-50257/2021	EPI_ISL_ 3274433	2021-07-18	Instituto Nacional de Investigación de Salud Pública - Centro de Referencia Nacional de Influenza y otros Virus Respiratorios	Instituto Nacional de Investigación de Salud Pública - Centro de Referencia Nacional de Influenza y otros Virus Respiratorios	Bruno et al.

Virus name	Accession number	Collected	Originating lab	Submitting lab	Author
hCoV-19/Brazil/SP-IB_146354/2021	EPI_ISL_ 8721492	2021-12-28	BIOFAST	Instituto Butantan	Covas et al.
hCoV-19/Brazil/RJ-LNN07279/2021	EPI_ISL_ 8056949	2021-12-01	Laboratório Central de Saúde Pública Noel Nutels	Laboratório de Bioinformática	Almeida et al.
hCoV-19/Brazil/MG-FIOCRUZ-361/2021	EPI_ISL_ 8630314	2021-11-19	Laboratório Municipal de Biologia Molecular	Instituto Rene Rachou - Fiocruz Minas	Silva et al.
hCoV-19/Brazil/AM-FIOCRUZ-ILMD2200227/2 021	EPI_ISL_ 8554924	2021-12-28	Laboratório Central de Saúde Pública do Amazonas	Laboratório de Ecologia de Doenças Transmissíveis na Amazônia, Instituto Leônidas e Maria Deane - Fiocruz Amazônia	Silva et al.
hCoV-19/Brazil/SC-NVBS7750GEN OV45616570561/2021	EPI_ISL_ 7366560	2021-10-04	DASA	DASA	Guarischi et al.
hCoV-19/Brazil/RJ-LNN07342/2021	EPI_ISL_ 8056982	2021-11-29	Laboratório Central de Saúde Pública Noel Nutels	Laboratório de Bioinformática	Almeida et al.
hCoV-19/Brazil/ES-FIOCRUZ-65360/2021	EPI_ISL_ 8332009	2021-10-28	Laboratório Central de Saúde Pública do Estado do Espírito Santo	Laboratório de Vírus Respiratórios e Sarampo, Instituto Oswaldo Cruz, Fundação Oswaldo Cruz	Resende et al.
hCoV-19/Brazil/BA-LACEN-BA954-291993290/2021	EPI_ISL_ 8590638	2021-12-13	Laboratório Central de Saúde Pública do Estado da Bahia	Laboratório Central de Saúde Pública do Estado da Bahia	Tosta et al.
hCoV-19/Brazil/SP-IB_136559/2021	EPI_ISL_ 5898397	2021-10-04	Inside Diagnósticos	Instituto Butantan	Covas et al.
hCoV-19/Brazil/PR-NVBS7226GEN OV8280977377 11/2021	EPI_ISL_ 6997471	2021-09-09	DASA	DASA	Guarischi et al.
hCoV-19/Brazil/MS-IAL6924/2021	EPI_ISL_ 7565753	2021-12-01	Laboratório Central de Saúde Pública do Estado de Mato Grosso do Sul	Instituto Adolfo Lutz, Centro Interdisciplinar de Procedimentos, Laboratório Estratégico	Sacchi et al.
hCoV-19/Brazil/CE-FIOCRUZ-14174/2021	EPI_ISL_ 5529874	2021-08-12	Núcleo de Enfrentamento à Covid-19	Laboratório de Epidemiologia Molecular de Competência Analítica - Fundação Oswaldo Cruz Ceará	Miyajima et al.
hCoV-19/Brazil/SP-IB_143461/2021	EPI_ISL_ 7808931	2021-11-28	Inside	Instituto Butantan	Covas et al.
hCoV-19/Brazil/SP-IB_137647/2021	EPI_ISL_ 6508861	2021-10-12	BIOFAST	Instituto Butantan	Covas et al.
hCoV-19/Brazil/SC-FIOCRUZ-56924/2021	EPI_ISL_ 6173534	2021-09-23	Laboratório Central de Saúde Pública do Estado de Santa Catarina	Laboratório de Vírus Respiratórios e Sarampo, Instituto Oswaldo Cruz, Fundação Oswaldo Cruz	Resende et al.
hCoV-19/Brazil/PR-FIOCRUZ-60281/2021	EPI_ISL_ 6772699	2021-10-26	Laboratório Central de Saúde Pública do Estado do Paraná	Laboratório de Vírus Respiratórios e Sarampo, Instituto Oswaldo Cruz, Fundação Oswaldo Cruz	Resende et al.
hCoV-19/Brazil/SP-IB_146511/2021	EPI_ISL_ 8721946	2021-12-28	BIOFAST	Instituto Butantan	Covas et al.
hCoV-19/Brazil/RJ-LNN07165/2021	EPI_ISL_ 7976284	2021-11-17	Laboratório Central de Saúde Pública Noel Nutels	Laboratório de Bioinformática	Almeida et al.
hCoV-19/Brazil/BA-LACEN-BA869-291931863/2021	EPI_ISL_ 6945670	2021-10-20	Laboratório Central de Saúde Pública do Estado da Bahia	Laboratório Central de Saúde Pública do Estado da Bahia	Tosta et al.
hCoV-19/Brazil/SP-IB_128475/2021	EPI_ISL_ 4168674	2021-08-23	UBS Glenda Rodella Dumas	Instituto Butantan	Covas et al.
hCoV-19/Brazil/SC-FIOCRUZ-54171/2021	EPI_ISL_ 5329002	2021-09-24	Laboratório Central de Saúde Pública do Estado de Santa Catarina	Laboratório de Vírus Respiratórios e Sarampo, Instituto Oswaldo Cruz, Fundação Oswaldo Cruz	Resende et al.
hCoV-19/Brazil/ES-FIOCRUZ-49165/2021	EPI_ISL_ 4220215	2021-08-14	Laboratório Central de Saúde Pública do Estado do Espírito Santo	Laboratório de Vírus Respiratórios e Sarampo, Instituto Oswaldo Cruz, Fundação Oswaldo Cruz	Resende et al.
hCoV-19/Brazil/AM-FIOCRUZ-ILMD2100728/2 021	EPI_ISL_ 5490895	2021-09-20	Laboratório Central de Saúde Pública do Estado do Amazonas	Laboratório de Ecologia de Doenças Transmissíveis na Amazônia, Instituto Leônidas e Maria Deane - Fiocruz Amazônia	Silva et al.
hCoV-19/Brazil/SP-IB_135451/2021	EPI_ISL_ 5689173	2021-09-28	BIOFAST	Instituto Butantan	Covas et al.
hCoV-19/Brazil/GO-FIOCRUZ-55484/2021	EPI_ISL_ 6172815	2021-08-31	Laboratório Central de Saúde Pública do Estado de Goiás	Laboratório de Vírus Respiratórios e Sarampo, Instituto Oswaldo Cruz, Fundação Oswaldo Cruz	Resende et al.
hCoV-19/Brazil/BA-LACEN-836-291730739/2021	EPI_ISL_ 6692552	2021-10-04	Laboratório Central de Saúde Pública do Estado da Bahia	Laboratório Central de Saúde Pública do Estado da Bahia	Tosta et al.
hCoV-19/Brazil/BA-LACEN-BA761-291682183/2021	EPI_ISL_ 5146240	2021-09-16	Laboratório Central de Saúde Pública do Estado da Bahia	Laboratório Central de Saúde Pública do Estado da Bahia	Tosta et al.
hCoV-19/Brazil/RR-FIOCRUZ-ILMD2200047/2 021	EPI_ISL_ 8360720	2021-10-04	Laboratório Central de Saúde Pública do Estado de Roraima	Laboratório de Ecologia de Doenças Transmissíveis na Amazônia, Instituto Leônidas e Maria Deane - Fiocruz Amazônia	Meneses et al.
hCoV-19/Brazil/AM-FIOCRUZ-ILMD2200241/2 021	EPI_ISL_ 8554937	2021-12-20	Laboratório Central de Saúde Pública do Amazonas	Laboratório de Ecologia de Doenças Transmissíveis na Amazônia, Instituto Leônidas e Maria Deane - Fiocruz Amazônia	Silva et al.
hCoV-19/Brazil/SP-IB_142457/2021	EPI_ISL_ 7665682	2021-11-18	Laboratório de Franca	Instituto Butantan	Covas et al.
hCoV-19/Brazil/SC-FIOCRUZ-65179/2021	EPI_ISL_ 8266382	2021-12-09	Laboratório Central de Saúde Pública do Estado de Santa Catarina	Laboratório de Vírus Respiratórios e Sarampo, Instituto Oswaldo Cruz, Fundação Oswaldo Cruz	Resende et al.
hCoV-19/Brazil/PR-FIOCRUZ-60232/2021	EPI_ISL_ 6980543	2021-10-21	Laboratório Central de Saúde Pública do Estado do Paraná	Laboratório de Vírus Respiratórios e Sarampo, Instituto Oswaldo Cruz, Fundação Oswaldo Cruz	Resende et al.
hCoV-19/Brazil/AL-IB_139215/2021	EPI_ISL_ 6570066	2021-09-22	Laboratório Central de Saúde Pública do Estado de Alagoas	Instituto Butantan	Covas et al.
